# Distinct factors explain life space mobility below and above the age of 75 years old in older adults

**DOI:** 10.1007/s11357-025-01873-6

**Published:** 2025-09-05

**Authors:** Samar Assadi Khalil, Efrat Gil, Roy Tzemah-Shahar, Faisal Azaiza, Rachel Kizony, Maayan Agmon

**Affiliations:** 1 Faculty of Social Welfare & Health Sciences, The Cheryl Spencer Department of Nursing, University of Haifa, Haifa, Israel; 2 Department of Occupational Therapy, Zefat Academic College, Zefat, Israel; 3 Division of Geriatrics, Israeli Ministry of Health, Jerusalem, Israel; 4 Faculty of Social Welfare and Health Sciences, School of Social Work, University of Haifa, Haifa, Israel; 5 Faculty of Social Welfare & Health Sciences, Department of Occupational Therapy, University of Haifa, Haifa, Israel

**Keywords:** Life space mobility, Older adults, Mobility, Cognition, Fear of falling

## Abstract

Life space mobility (LSM) is important for participation in daily life. It is influenced by individual and environmental factors and tends to decline with age. Although LSM has been studied in older adults, stratification of this population into age subgroups has not been performed, creating a gap in understanding the factors associated with LSM in a more granular manner. This cross-sectional study aimed to identify the factors associated with LSM in community-dwelling older adults below and above the age of 75. Participants aged 65 and older without neurological conditions or dementia were recruited. LSM was assessed using the Life Space Assessment (LSA), mobility with the Timed Up and Go test (TUG), cognition with the Montreal Cognitive Assessment (MoCA), fear of falling with the Activities-specific Balance Confidence scale (ABC), and depression with the Hospital Anxiety and Depression Scale (HADS). Additional self-reported data included employment/volunteering, frequency of leaving the house, functional status, and number of medications. Separate regression models were conducted for each age subgroup. Two-hundred forty-two older adults (28.9% men) were recruited (mean (SD) age 73.7(6.4) years), with 40.9% aged over 75. In the younger subgroup, sex, frequency of leaving the house, TUG, and employment/volunteer status significantly explained 42.8% of the variance in LSM. In the older subgroup, sex, age, ABC, MoCA, and TUG significantly explained 46.9% of the variance in LSM. Distinct factors are associated with LSM in each age subgroup. Accordingly, future interventions should be tailored for each subgroup individually.

## Introduction

Between 2021 and 2050, the percentage of the global population aged 65 and older is expected to increase significantly from less than 10% to about 17% [1]. In light of this projected demographic shift, ensuring the quality of life of older adults is a critical public health objective [2]. Mobility is considered one of the most important factors associated with quality of life. As age increases, mobility difficulties are common, due to deterioration in gait, balance, and lower extremity functions [3]. Limited mobility is linked to functional decline, higher mortality rates, and escalated healthcare expenses [4].

Life space mobility (LSM), a spatial assessment of mobility patterns in daily life, provides a lens into an individual’s actual mobility and his access to various community amenities. LSM reflects the opportunities for participating in diverse life events beyond the home [2]. A higher LSM is linked to an enhanced sense of well-being through participation in a variety of daily activities [5]. Conversely, a lower LSM signifies reduced participation in social activities, contributing to a diminished quality of life and overall well-being [5]. Furthermore, a decline in LSM is associated with adverse health outcomes, including falls, fractures, frailty development, institutionalization, and even mortality [6].

Moreover, limited LSM is associated with a decline in cognitive abilities among older adults. In a comprehensive systematic review, lower LSM was correlated with diminished executive function, learning memory, and processing speed [6]. Similarly, a large cohort study conducted on 1,684 community-dwelling men aged 65 and over [mean (SD) age 77.1 (4.2) years] revealed that a decrease in LSM is linked to accelerated cognitive decline (assessed using the Modified Mini-Mental State (3MS) Test, and Trail-Making Test Part B) and an increased incidence of dementia over a 7-year follow-up period [7].

Previous studies have explored the factors linked to declining LSM in older adults. For instance, a cross-sectional study [5] involving 3500 community-dwelling older adults aged 65 and over, with a mean age of 73.5 (6.1) years, revealed associations between LSM and locomotion, depression, health literacy, participation, and social support. In the same vein, another large cross-sectional study encompassing 12,646 older adults aged 65 and over (mean age 73.0 (5.7) years) identified driving, social support, and walking speed as the most significant variables correlated with LSM [8]. However, it is worth noting that all previous studies treated their study populations as a homogeneous group, and differences between age subgroups were ignored. Categorizing older adults into age subgroups is imperative since age significantly influences health status [9]. As individuals age, the risk of health conditions such as dementia, falls, and chronic diseases increase [10, 11], as does the likelihood of experiencing multiple conditions simultaneously [11]. Indeed, these findings underscore the importance of considering age-specific factors when studying LSM and the factors associated with it in older adults.

Therefore, the primary aim of our study is to delve into the factors associated with LSM in older adults within specific age subgroup. We follow the categorization proposed by Alterovitz et al., who divided individuals into three age subgroups: the youngest-old (aged 65–74 years), the middle-old (aged 75–84 years), and the oldest-old (aged over 85 years) [12]. However, given the limited number of participants in the oldest-old subgroup (*n* = 15), this group was merged with the middle-old group for analytical purposes. Thus, our sample was categorized into two age subgroups: older adults less than 75 years old were the “younger subgroup” and older adults 75 years and older were the “older subgroup,” with age 75 selected as a meaningful cut-off point based on accumulating evidence highlighting this age as a turning point in late adulthood. Previous studies have used it to differentiate between younger-old and older-old adults, noting increased prevalence of multi-morbidity, cognitive decline, and mobility limitations beyond this age [4]. Recent evidence also suggests that age 75 marks a biological tipping point, where both resilience (the capacity to recover from health deficits) and robustness (resistance to decline) sharply decrease, resulting in accelerated health deterioration and increased mortality risk [13].

The purpose of the division into age subgroups is to provide insights into the unique factors associated with LSM at different stages of aging. By understanding these factors within each age subgroup, we can tailor primary preventative strategies to enhance LSM and overall well-being.

## Methods

### Study design

This study analyzed data derived from a larger cross-sectional study. These data have not been previously published or analyzed elsewhere. Two separate regression models were constructed for the younger and older subgroups, respectively, to understand the factors associated with LSM in each of the two age subgroups.

### Study population

We recruited older adults who met the following inclusion criteria: (1) aged 65 years and above, (2) community-dwelling, and (3) understand simple instructions, based on the assessor’s impression. The exclusion criteria were adults with self-reported neurological condition or dementia. This study was approved by the local Ethics Committee; all participants provided written informed consent prior to participation.

### Tools

The dependent variable, LSM, was assessed using the Life Space Assessment (LSA) [14]. Participants were asked if they have been in the following five life-space levels within the past month: at home beyond the room in which they sleep (level 1); in the area immediately outside their home, such as a porch or deck (level 2); beyond their own property or apartment building in the immediate neighborhood (level 3); outside their immediate neighborhood but within their town (level 4); and outside their immediate town (level 5). For each life-space level, participants were also asked how often they traveled to that area and whether they needed an assistive device or assistance from another person. LSA scores range from 0 (mobility confined to one’s bed) to 120 (traveled out of town every day without personal assistance or an assistive device). A higher score indicates higher LSM.

The independent variables were assessed using the following clinical assessments: The Timed Up and Go test (TUG) [15] is a screening tool to assess mobility and balance. Participants were asked to stand up from a sitting position, walk 3 m, circumvent a static object, return, and adopt the initial position, and the time taken to complete the task was recorded. A cut-off score of 13.5 s has been shown to predict falling in older community-dwelling adults [16].

The Activities-specific Balance Confidence Scale (ABC) [17] assesses an individual’s fear of falling while performing 16 activities (for example, walking up or down stairs, standing on a chair to reach for something, sweeping the floor, walking around the house). Participants were asked to rate their confidence in completing each of the activities without losing their balance on a scale from 0% (no confidence) to 100% (completely confident). The total score is calculated by summing all the activities’ scores and dividing the total by 16. This total score ranges from 0 to 100%.

The Montreal Cognitive Assessment (MoCA) [18] is a screening test to assess the following cognitive domains: attention and concentration, executive functions, memory, language, visuo-constructional skills, conceptual thinking, calculations, and orientation. Score ranges from 0 (severe cognitive impairment) to 30 (normal cognitive ability).

The depression component of the Hospital Anxiety and Depression Scale (HADS) [19] is a self-report questionnaire that assesses different aspects of depression over the last week (for example, I still enjoy the things I used to enjoy; I can laugh and see the funny side of things). Each of the seven depression items is rated on a 4-point scale ranging from 0 (yes, definitely) to 3 (no, not at all). The total score ranging from 0 to 21, and a higher score indicates higher depression.

In addition, we collected self-reported data on each individual’s employment or volunteer commitments (work or volunteer = 1, no = 0) and how frequently they left the house each week. Participants were also asked about their ability to independently complete basic activities of daily living (BADL), and demographic information as well as the number of medical diagnoses and medications was recorded.

### Procedure

In the first stage of the study, a telephone conversation was held with eligible adults to provide an oral explanation regarding the study. Then data collection was performed in the participant’s home by a health professional (e.g., occupational therapist or nurse).

### Statistical analysis

Descriptive statistics were used to describe the study population and the two age subgroups, considering demographic information, cognition, mobility, fear of falling, and LSM. Since most of the variables were distributed normally, the data are presented as the mean and SD, and parametric comparative statistic tests were used. Missing data were handled as follows: for variables with only 1–2 missing values (frequency of leaving home per week and depressive symptoms), the mean value of the variable was used to replace the missing cases. For variables with more substantial missingness (TUG, 24 cases; MoCA, 15 cases), multiple imputation was applied [20]. SPSS generated the original model and five models after imputation; no pooled model was available. Results were consistent across the imputed models, with only minimal differences. We have also refined the description of the imputation process in the Data Analysis section to clarify the rationale and procedures used. One of the imputed models was selected for presentation in the manuscript as it was most similar to the original model and ensures reliable findings. Additionally, for frequency of leaving the home per week, all values greater than 8 were recorded as 8 to account for potential outliers and ensure consistency in the analysis. This cap was chosen to reflect a typical upper bound for community-dwelling independent older adults, corresponding approximately to one outing per day, which aligns with common mobility patterns in this population [21].

Pearson correlation tests were used to assess correlations between LSM and the continuous independent variables including age, mobility, fear of falling, independence in BADL, frequency of leaving home per week, cognition, symptoms of depression, and number of medications and diagnoses, for all study population and for the two age subgroups separately. For the categorical variables, sex, and employment or volunteer status, independent-sample *t*-test were conducted. Variables that had a significant correlation (*p* < 0.05) with LSM were included in a multiple regression model using a forward stepwise method. We used this method to select the best independent variables for inclusion in the final model [22]. Multi-collinearity was assessed using variance inflation factors (VIF), with all values below 5, indicating no problematic correlations among predictors. Two separate regression models were constructed for older adults below and above the age of 75 years, respectively*.*

## Results

Data were collected between Sep 2021 and Aug 2023. A total of 242 older adults (28.9% men) were eligible and agreed to participate (the mean age was 73.7 (5.6) years and 11.8 (4.3) years of education). Of these, 99 participants (40.9%) were 75 or older. This older subgroup had a mean LSA score of 79.8 (19.8), MoCA score of 21.1 (4.2), and TUG test completion time of 14.2 (6.4) sec. In contrast, the younger subgroup had a mean LSA 70.1 (20.5), had a MoCA score of 23.4 (3.7), and completed the TUG in 11.4 (5.0) s. See Table 1 for a comparison of additional demographic, cognitive, mobility, and fear of falling measures across the two age subgroups.

**Table 1:** Characteristics of the study population, comparing older adults divided into two age subgroups: younger than 75 years and 75 years or older

	Study population (*N* = 242)	Older adults < 75 (*N* = 143)	Older adults ≥ 75 (*N* = 99)	*t*-test
	Mean (SD)	Mean (SD)	Mean (SD)	*t*, *p*
Age (years)	73.7 (5.6)	70.0 (2.7)	79.1 (4.0)	− 19.76, < 0.001
Education (years)	11.8 (4.3)	12.0 (4.1)	11.4 (4.6)	1.07, 0.29
MoCA (0–30)	22.5 (4.0)	23.4 (3.7)	21.1 (4.2)	4.29, < 0.001
TUG (s)	12.5 (5.8)	11.4 (5.0)	14.2 (6.4)	− 3.54, < 0.001
ABC (0–100%)	74.5 (22.0)	77.3 (19.1)	70.6 (25.2)	2.23, 0.03
LSA (0–120)	75.8 (20.6)	79.8 (19.8)	70.1 (20.5)	3.70, < 0.001
HADS (depression part)	5.86 (2.35)	5.82 (2.30)	5.91 (2.42)	− 0.32, 0.75
Frequency of leaving home per week	5.4 (2.1)	5.6 (1.9)	5.1 (2.1)	1.85, 0.06
	N (%)	N (%)	N (%)	X^2^, *p*
Sex (M/F)	70 (28.9)/172 (71.1)	39 (27.3)/104 (72.7)	31 (31.3)/68 (68.7)	0.47, 0.496
Employment or volunteer status (Y/N)	116 (47.9)/126 (52.1)	79 (55.2)/64 (44.8)	37 (37.4)/62 (62.6)	7.48, 0.006

*MoCA* the Montreal Cognitive Assessment, *TUG* Timed UP and Go Test, *ABC* the Activities-specific Balance Confidence scale, *LSA* Life Space Assessment, *HADS* the Hospital Anxiety and Depression Scale

In the younger subgroup, the mean (SD) of LSA was 87.14 (16.62) for men and 77.06 (20.24) for women with a significant difference between sexes (*p* = 0.003). In the older subgroup, the mean (SD) was 78.37 (19.97) for men and 66.32 (19.97) for women, with a significant difference as well (*p* = 0.006) (see Table 2).

**Table 2:** Differences in life space mobility, assessed with the life space assessment, between sexes in both subgroups

	Older adults < 75 (*N* = 143)			Older adults ≥ 75 (*N* = 99)		
	Male (*N* = 39)	Female (*N* = 104)	*t-test*	Male (*N* = 31)	Female (*N* = 68)	*t*-test
	Mean (SD)	Mean (SD)	*t*, *p*	Mean (SD)	Mean (SD)	*t*, *p*
LSA (0–120)	87.14 (16.62)	77.06 (20.24)	2.83, 0.003	78.37 (19.97)	66.32 (19.79)	2.57, 0.006

*LSA* Life Space Assessment

In the younger subgroup, LSM was significantly associated with sex, mobility, fear of falling, employment or volunteer status, frequency of leaving the home per week, cognition, symptoms of depression, and the number of medications. In the older subgroup, LSM was significantly correlated with age, sex, mobility, fear of falling, employment or volunteer status, frequency of leaving home per week, cognition, symptoms of depression, and the number of medications and diagnoses (see Table 3).

**Table 3:** Pearson correlations and group comparisons (*t-*test) between Life Space Assessment and independent variables for the entire study population and for each age subgroup separately

	Study population (*N* = 242)	Older adults < 75 (*N*= 143)	Older adults ≥ 75 (*N* = 99)
	LSA *R*	LSA *r*	LSA *r*
**Age**	− 0.27**	− 0.07	− 0.20*
**TUG**	− 0.53**	− 0.48**	− 0.53**
**ABC**	0.48**	− 0.35**	0.59**
**Independence in BADL**	0.32**	− 0.15	− 0.38**
**Frequency of leaving home per week**	0.47**	0.49**	0.42**
**MoCA**	0.29**	0.19*	0.30**
**HADS (depression part)**	− 0.29**	− 0.32**	− 0.25**
**Number of diagnoses**	− 0.21**	− 0.16	− 0.21*
**Number of medications**	− 0.30**	− 0.19*	− 0.33**
	*t*-test	*t*-test	*t*-test
**Sex**	3.66**	3.04*	2.80*
**Employment or volunteer status**	− 7.137**	− 5.34**	− 3.82**

*LSA* Life Space Assessment, *TUG* Timed UP and Go test, *ABC* the Activities-specific Balance Confidence scale, *MoCA* the Montreal Cognitive Assessment, *HADS* the Hospital Anxiety and Depression Scale

*p < 0.05; **p < 0.01

In a stepwise regression model constructed for the younger subgroup, the variance in LSM explained by sex, frequency of leaving home per week, mobility, and employment or volunteer status was 42.8% (5%, 20.7%, 12.7%, and 4.2% respectively) and all contributions were significant. In the model constructed for the older subgroup, sex, age, fear of falling, cognition, and mobility explained 46.5% variance in LSM (7.5%, 4.5%, 27.7%, 3.5%, and 3.2% respectively) and all contributions were significant (see Table 4).

**Table 4:** Regression models used to identify the factors which contribute to life space mobility for older adults below 75 years and for those 75 years and older, respectively

	*R*^2^	*R*^2^_Change_	*B* (SE)	*Β*	*P*
Older adults < 75 years					
Sex	0.05	0.05	− 10.08 (3.63)	− 0.23	0.006
Frequency of leaving home per week	0.26	0.207	4.68 (0.75)	0.47	< 0.001
TUG	0.387	0.127	− 1.43 (0.27)	− 0.37	< 0.001
Employment or volunteer status	0.428	0.042	9.04 (2.84)	0.29	0.002
Older adults ≥ 75 years					
Sex	0.075	0.075	− 12.05 (4.30)	− 0.27	0.006
Age	0.12	0.045	− 1.08 (0.49)	− 0.21	0.028
ABC	0.398	0.277	0.44 (0.07)	0.54	< 0.001
MoCA	0.433	0.035	0.94 (0.39)	0.20	0.018
TUG	0.465	0.032	− 0.70 (0.30)	− 0.21	0.021

*TUG* Timed UP and Go test, *ABC* the Activities-specific Balance Confidence scale, *MoCA* the Montreal Cognitive Assessment

## Discussion

Our findings reveal that distinct factors are associated with LSM in two age subgroups of older adults: those below 75 years and those 75 years and above. While employment or volunteer status and frequency of leaving home per week explained a large portion of the variance in LSM in the younger subgroup, age, fear of falling, and cognitive decline were the most significant determinants in the older subgroup. Despite these differences, mobility and sex emerged as a key factor explaining LSM across both groups.

Previous studies have identified participation, depression, locomotion [5], social support [5, 8], walking speed, and driving [8], as key variables associated with LSM. However, these studies typically treated all older adults as a single group, ranging from 65 to 100 years [5] or 65 to 94 years [8]. This broad categorization may have obscured important age-related differences that could interact with or confound LSM, such as cognitive decline, increased fall risk, and the prevalence of multiple comorbidities. Our study addresses this gap by analyzing LSM determinants separately in two distinct age subgroups.

In the younger subgroup, employment or volunteer status, mobility, and frequency of leaving the house per week emerged as key factors explaining the variance in LSM. Research suggests that individuals aged 67–74 are the most active volunteers [23], emphasizing their continued engagement in social and professional roles. These activities inherently require individuals to leave their homes, navigate diverse environments, and interact with their communities, all of which contribute to expanded LSM.

The positive impact of employment and volunteering extends beyond mobility. Engaging in these activities fosters a sense of purpose, social connections, and cognitive stimulation, all of which contribute to well-being in later life [24]. Given that volunteering is relatively common in this age range, with participation rates reaching up to 42% among those aged 65 and over, and up to 47% among those aged 60 and over [24], initiatives promoting accessible volunteer opportunities could serve as a valuable strategy for maintaining LSM and supporting active aging.

In contrast, fear of falling, cognitive decline, mobility, and age were the most significant predictors of LSM in the older subgroup. These findings align with prior research highlighting age-related changes that contribute to reduced mobility and participation in various aspects of life [10, 11]. However, older adults are not a homogeneous group, and the impact of these factors may vary significantly across different stages of later life. This underscores the importance of analyzing subgroups separately, as done in the present study. Fear of falling, for instance, becomes increasingly prevalent with advancing age [25] and is associated with activity restriction, mobility, social withdrawal, depression, and diminished quality of life [26, 27]. This creates a negative cycle, where reduced mobility further exacerbates cognitive decline, as evidenced by studies linking mobility with cognitive function [3], executive function [28], and overall cognitive performance [29]. These patterns highlight distinct challenges within the older subgroup, reinforcing the need for age-sensitive interventions that address mobility barriers and cognitive vulnerabilities in late life.

The variability in explanatory factors across the two age subgroups supports the commonly used division of older adults into subgroups, with age 75 as meaningful cut-off. These findings suggest that the factors associated with LSM differ significantly between older adults aged 65–74 and those aged 75 and older, reflecting the distinct functional and health-related needs of each group. This classification allows for the identification of age-specific requirements and provides a foundation for developing more tailored strategies for intervention and support.

Sex, along with mobility, was among the explanatory factors associated with LSM in both age subgroups. Consistent with prior research, our results indicate that men and women experience LSM differently, likely due to differences in social roles, health status, and risk perception [8]. Women, for instance, may have lower LSM due to higher levels of fear of falling [30] and lower participation in employment or driving [31], while men may be more affected by the transition out of work life. These sex-related differences highlight the need for tailored interventions that address specific barriers for men and women across age subgroups. Furthermore, our findings resonate with the sex health paradox, the well-documented phenomenon whereby women tend to live longer than men but experience higher rates of morbidity and disability [32]. These health disadvantages may contribute to reduced LSM in later life, particularly among older women.

### Limitations

This study had several limitations. First, there was limited representation of participants aged 85 and above. Due to the small number of participants in this age class, the sample was divided into only two age subgroups for analysis. With larger sample sizes, future studies could further stratify participants into three age groups, providing a deeper understanding of factors associated with LSM in adults aged 85 and above. Second, data regarding proprioception, which tends to decline with age [33] and may be associated with LSM, was not collected in this study. Future research should consider incorporating assessments of sensory status to better understand its relationship with LSM in older adults. Third, the sex distribution was not equal within age subgroups, with women being more represented than men. Given that sex differences influence LSM, future studies should explore the potential impact of sex on LSM and consider strategies to ensure more balanced representation. Fourth, although the variable “frequency of leaving the home” may indirectly capture aspects such as transportation access or community participation, the study did not include direct measures of psychosocial or environmental factors (e.g., anxiety, social support, neighborhood walkability), which are known to influence LSM. In addition, participants in this study had relatively homogeneous socioeconomic status and neighborhood characteristics, which may have limited the ability to detect associations with these factors. Future research should incorporate these dimensions more explicitly and examine them in more socioeconomically and environmentally diverse populations.

## Conclusions

Our findings emphasize the importance of considering age-specific factors when designing interventions to promote LSM. Employment and volunteer engagement and frequency of leaving the home appear to be key facilitators of LSM in the younger subgroup, while fear of falling, cognitive decline, and advanced age become more dominant barriers in the older subgroup. While age itself is not modifiable, recognizing these age-related patterns allows for the development of subgroup-specific interventions that respond to the distinct risk factors of individuals across different life stages. Importantly, while many previous studies have examined LSM in older adults, they always treated this population as a homogeneous group, overlooking critical differences that emerge across age segments (e.g., 65–74 vs. 75 +). By explicitly distinguishing between two age subgroups, our study contributes a more nuanced understanding of how determinants of LSM shift over the aging process. Additionally, sex was found to be a significant factor influencing LSM across both age groups. By addressing the interplay between mobility, cognitive, and social engagement, interventions can be tailored to meet the unique needs of older adults at different stages of aging.

## Data Availability

The study data are available from the corresponding author upon request.
